# Rapid peat accumulation favours the occurrence of both fen and bog microbial communities within a Mediterranean, free-floating peat island

**DOI:** 10.1038/s41598-017-08662-y

**Published:** 2017-08-17

**Authors:** Giuseppe Concheri, Piergiorgio Stevanato, Claudio Zaccone, William Shotyk, Valeria D’Orazio, Teodoro Miano, Pietro Piffanelli, Valeria Rizzi, Chiara Ferrandi, Andrea Squartini

**Affiliations:** 1 Department of Agronomy, Animals, Natural Resources and Environment - DAFNAE, University of Padua, Viale dell’Università 16, 35020 Legnaro (Padova), Italy; 2 Department of the Sciences of Agriculture, Food and Environment, University of Foggia, via Napoli 25, 71122 Foggia, Italy; 3 Department of Renewable Resources, University of Alberta, 348B South Academic Building, T6G 2H1 Edmonton, Canada; 4 Department of Soil, Plant and Food Sciences, University of Bari “Aldo Moro”, via Amendola 165/A, 70126 Bari, Italy; 5 Parco Tecnologico Padano Foundation, via Einstein 1, Cascina Codazza, 26900 Lodi, Italy

## Abstract

The unique environment of a 4m-thick, free-floating peat island within the Posta Fibreno lake (Central Italy) was analyzed using DNA-based techniques to assess bacterial and fungal community members identity and abundance. Two depths were sampled at 41 and 279 cm from the surface, the former corresponding to an emerged portion of *Sphagnum* residues accumulated less than 30 yrs ago, and the latter mainly consisting of silty peat belonging to the deeply submerged part of the island, dating back to 1520–1660 AD. The corresponding communities were very diverse, each of them dominated by a different member of the Delta-proteobacteria class for prokaryotes. Among Eukaryotes, Ascomycota prevailed in the shallow layer while Basidiomycota were abundant in the deep sample. The identity of taxa partitioning between acidic surface layer and neutral core is very reminiscent of the differences reported between bogs and fens respectively, supporting the view of Posta Fibreno as a relic transitional floating mire. Moreover, some microbial taxa show an unusual concurrent species convergence between this sub-Mediterranean site and far Nordic or circumpolar environments. This study represents the first report describing the biotic assemblages of such a peculiar environment, and provides some insights into the possible mechanisms of its evolution.

## Introduction

Free-floating mires are particular environments consisting of emergent vegetation rooted in highly organic buoyant mats, moving on the lake surface naturally due to the release of gases generated by the decomposition of the underlying peat-mat layer^1^. Free-floating mires normally show a thickness ranging from few tens to 250 cm, depending mainly on below-ground biomass allocation, morphology of the constituent plant species and hydrology^2, 3^. The aspect of wandering islands has stirred human curiosity since antiquity^4^.

**Figure 1:**
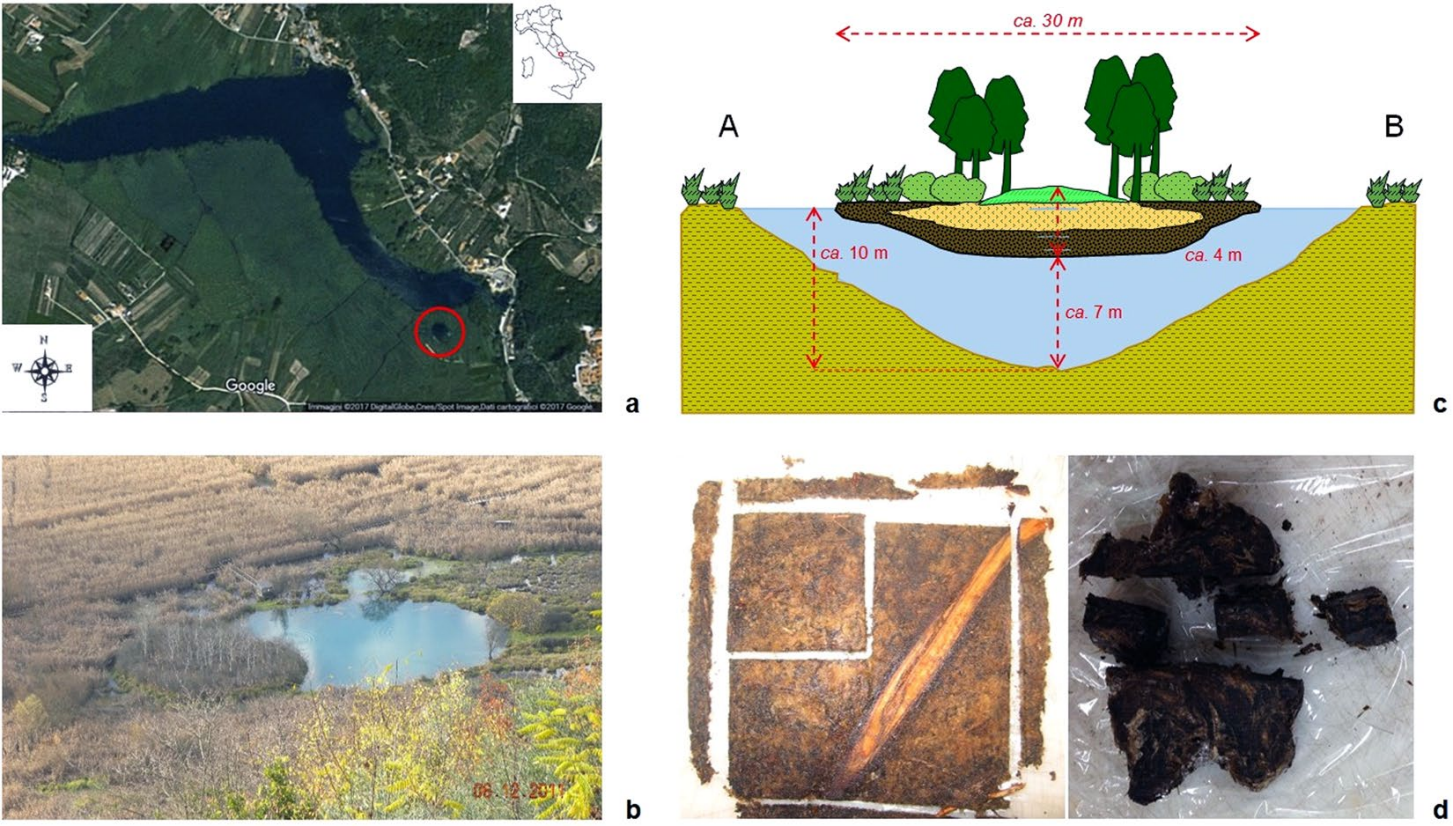
The Posta Fibreno floating island (“la Rota”). (**a**) Aerial view of the Posta Fibreno lake (source: Image ©2017 DigitalGlobe, Cnes/Spot Image, Dati cartografici ©2017 Google), with a red circle surrounding the floating island. The map in the top right corner was created using QGIS v. 2.14 software (http://www.qgis.org/it/site/). (**b)** The Posta Fibreno free-floating island (photo by C.Z.). (**c)** Schematic representation of the studied floating mire (drawn by C.Z). (**d)** The shallow (left) and deep (right) peat samples (photos by C.Z.).

The free-floating island of Posta Fibreno, known as “la Rota” (“*the wheel*”), is one of the few surviving in southern Europe, and, to the best of our knowledge, represents the thickest accumulation of floating peat ever documented in the scientific literature^5^. The site is part of a Natural Reserve appointed as Special Area of Conservation (SAC-IT6050015) due to the presence of habitats and species (92/43/EEC) of prominent scientific and naturalistic value. The relic mire of Posta Fibreno supports a vegetation of *Carex paniculata* reeds, *Sphagnum palustre* and sparse trees including willows (*Salix cinerea*) and poplars (*Populus tremula*)^6^. The island has a diameter of about 30 m and a maximum thickness of 4 m, 3 m of which lie below the water level; a 7-m water column separates the bottom of the island from the bottom of the lake (Fig. 1a–c).

With 400 cm of peat accumulated in less than 700 yr (624 ± 21 yr BP; Cal AD 1293–1396), resulting in an average growth rate of 0.5–0.6 cm/yr, the Posta Fibreno free-floating island shows highly anomalous accumulation rates of organic carbon (C) and total nitrogen (N) compared to many other peatland ecosystems^5^.

The free-floating island at Posta Fibreno is a transitional mire, where ombrotrophic bog vegetation has evolved over time from minerotrophic fen vegetation probably as a consequence of buoyancy^5^. While this is the most common evolutionary sequence found in continental peatlands that have formed from terrestrialization, almost no data are present in literature on the evolution of deep floating islands.

Here, we characterized the microbiological communities of two peat samples collected from this floating mire at different depths (41 and 279 cm) and which displayed different physical and chemical properties, botanical composition and age of peat accumulation (Fig. 1d). DNA was extracted from the two peat layers and analyzed by RealTime PCR and Next Generation Sequencing (NGS) approaches in order to unravel its unique evolution mechanisms and provide some evidences about microbial adaptation to environmental changes over centuries.

## Results

### RealTime PCR quantitative analysis of the N cycle genes

The results are shown in Table 1. The ammonia monooxygenase gene (*amoA*), catalyzing the first step of nitrification (conversion of ammonia NH_4_^+^ to hydroxylamine), was sought in two versions; archeal and bacterial, the former of which was below detectable levels, whereas the second was more abundant in the deeper (PFB4 #16–17, hereafter referred to as ‘deep’) sample as it appeared two cycles earlier when compared to the superficial one (PF2 #46, hereafter referred to as ‘shallow’). The two genes of the denitrification pathway, nitrite reductase *nirK* (nitrite to nitric oxide) and nitrous oxide reductase *nosZ* (nitrous oxide to N_2_), were also more abundant in the lower sample and so was the N fixation nitrogenase unit *nifH* (from N_2_ to NH_4_^+^).

**Table 1:** Quantitative PCR results. Data show the RealTime threshold cycles recorded for the different amplifications. Data are the means of three technical replicates ± Standard Deviation.

Sample	Sample code	Depth (cm)	*amoA* archaea	*amoA* eubacteria	*nirK*	*nosZ*	*nifH*
Shallow	PF2 #46	41	Undetectable	32.29 ± 0.19	24.58 ± 0.31	31.30 ± 0.26	32.75 ± 0.71
Deep	PFB4 #16–17	279	Undetectable	30.61 ± 0.24	23.81 ± 0.22	27.89 ± 0.37	30.47 ± 0.96

### 16S DNA sequencing

The analysis of the species-diagnostic amplified prokaryotic target region yielded 1784302 sequences from shallow sample (41 cm of depth) and 2388312 sequences from the deep sample (279 cm of depth). After the annotation procedure, the number of Operational Taxonomic Units (OTUs) at genus rank level for the two samples was 1738 and 2026, respectively. These encompassed a total of 62 bacterial or archaeal phyla. Table 2 shows the proportional abundance distribution of all phyla accounting for a frequency higher than 1% in at least one of the two samples. For the whole list refer to Supplementary material dataset S1, spreadsheet, taxa abundance list.

**Table 2:** Prokaryotic OTUs percent abundance distribution at phylum level. The superkingdom (bacteria or Archaea) is indicated, followed by the Phylum denomination. Taxa whose presence in at least one of the two samples was >1% are shown. Frequencies are ordered in decreasing abundance of the shallow sample column.

Phylum	Shallow sample	Deep sample
Bacteria; Proteobacteria	35.598	20.969
Bacteria; Acidobacteria	27.104	9.194
Bacteria; Verrucomicrobia	13.952	1.966
Bacteria; Chlorobi	5.767	2.232
Bacteria; Chloroflexi	3.743	17.515
Bacteria; Planctomycetes	2.242	1.864
Bacteria; Spirochaetes	1.928	4.839
Bacteria; Chlamydiae	1.818	0.147
Bacteria; Actinobacteria	1.562	1.671
Bacteria; Firmicutes	1.425	9.875
Bacteria; Bacteroidetes	1.138	5.230
Bacteria; TM6	0.702	0.392
Bacteria; Nitrospirae	0.677	4.880
Archaea; Thaumarchaeota	0.328	2.596
Archaea; Euryarchaeota	0.291	3.254
Bacteria; Candidate_division_OD1	0.094	3.409
Bacteria; Candidate_division_OP3	0.059	1.721
Bacteria; Fibrobacteres	0.033	1.024
Bacteria; TA06	0.008	1.839

As regards the finer taxonomic ranks resolution, the top scoring taxa (characterizing each of the two sampling depths as dominant in terms of percent abundance) are shown in Table 3 (sorted by abundance of the shallow sample and featuring those present at frequencies >1% in that sample and their corresponding levels in the deeper one) and in Table 4 (sorted by abundance of the deep sample and featuring those present at frequencies >1% in that sample and their corresponding levels in the shallow one). The whole list, containing the total 2267 taxa identified, is available in the Supplementary Material dataset S1, spreadsheet, taxa abundance list.

**Table 3:** Prokaryotic OTUs % at genus level (with cutoff >1%) sorted by abundance in surface sample.

OTUs	Shallow sample	Deep sample
Proteobacteria; Deltaproteobacteria; Syntrophobacterales; Syntrophaceae; Desulfobacca	7.022	0.132
Verrucomicrobia; Opitutae; Opitutales; Opitutaceae; Opitutus	6.845	0.271
Acidobacteria; Acidobacteria; Acidobacteriales; Acidobacteriaceae	6.286	0.158
Chlorobi; Ignavibacteria; Ignavibacteriales; BSV26	5.287	1.831
Acidobacteria; Acidobacteria; DA052	5.074	1.144
Acidobacteria; Acidobacteria; KF-JG30-18	4.302	3.207
Acidobacteria; Acidobacteria; Candidatus_Solibacter	4.208	0.357
Verrucomicrobia; OPB35_soil_group	3.776	1.003
Acidobacteria; Acidobacteria; Order_Incertae_Sedis; Family_Incertae_Sedis; Bryobacter	3.098	0.317
Verrucomicrobia; S-BQ2-57_soil_group	2.919	0.575
Proteobacteria; Alphaproteobacteria; Rhizobiales; alphaI_cluster	2.385	0.120
Proteobacteria; Alphaproteobacteria; Rhizobiales; Methylocystaceae; Methylocystis	1.940	0.012
Spirochaetes; Spirochaetes; Spirochaetales; Spirochaetaceae; Spirochaeta	1.901	3.728
Proteobacteria; Alphaproteobacteria; Rhizobiales; Xanthobacteraceae; Pseudolabrys	1.832	0.016
Proteobacteria; Alphaproteobacteria; Rhizobiales; Methylocystaceae	1.786	0.011
Proteobacteria; Alphaproteobacteria; Rhodospirillales; Acetobacteraceae	1.709	0.010
Proteobacteria; Alphaproteobacteria; Rhizobiales; Xanthobacteraceae	1.647	0.046
Proteobacteria; Deltaproteobacteria; Syntrophobacterales; Syntrophaceae; Smithella	1.584	0.413
Chloroflexi; KD4-96	1.521	0.618
Proteobacteria; Deltaproteobacteria; Syntrophorhabdaceae; Syntrophorhabdus	1.383	0.492
Chlamydiae; Chlamydiae; Chlamydiales; cvE6	1.338	0.060
Proteobacteria; Alphaproteobacteria; Rhizobiales; Beijerinckiaceae; Methylovirgula	1.256	0.006
Acidobacteria; Acidobacteria; Acidobacteriales; Acidobacteriaceae; Candidatus_Koribacter	1.164	0.020
Proteobacteria; Deltaproteobacteria; Syntrophobacterales; Syntrophobacteraceae; Syntrophobacter	1.138	0.238
Proteobacteria; Alphaproteobacteria; Rhodospirillales; DA111	1.101	0.025
Planctomycetes; Phycisphaerae; WD2101_soil_group	1.031	0.022

**Table 4:** Prokaryotic OTUs % at genus level (with cutoff >1%) sorted by decreasing abundance in the 280 cm-deep sample.

**OTUs**	Shallow sample	Deep sample
Proteobacteria; Deltaproteobacteria; Sva0485	0.022	7.588
Chloroflexi; Anaerolineae; Anaerolineales; Anaerolineaceae	0.882	5.236
Spirochaetes; Spirochaetes; Spirochaetales; Spirochaetaceae; Spirochaeta	1.901	3.728
Nitrospirae; Nitrospira; Nitrospirales; OPB95	0.007	3.532
Candidate_division_OD1	0.094	3.409
Acidobacteria; Acidobacteria; KF-JG30-18	4.302	3.207
Firmicutes; Bacilli; Bacillales; Bacillaceae; Bacillus	0.003	2.696
Bacteroidetes; vadinHA17	0.020	2.626
Archaea; Thaumarchaeota; Miscellaneous_Crenarchaeotic_Group	0.203	2.530
Chloroflexi; vadinBA26	0.068	2.512
Chloroflexi; GIF3	0.012	2.328
Chloroflexi; GIF9	0.388	2.157
Proteobacteria; Deltaproteobacteria; Syntrophobacterales; Syntrophaceae	0.080	2.053
Firmicutes; Clostridia; Clostridiales; Clostridiaceae; Clostridium	0.055	2.051
Bacteria; TA06	0.008	1.839
Chlorobi; Ignavibacteria; Ignavibacteriales; BSV26	5.287	1.831
Proteobacteria; Alphaproteobacteria; Sphingomonadaceae; Novosphingobium	0.020	1.762
Candidate_division_OP3	0.059	1.721
Acidobacteria; Acidobacteria; BPC102	0.203	1.502
Chloroflexi; MSBL5	0.000	1.147
Acidobacteria; Acidobacteria; DA052	5.074	1.144
Verrucomicrobia; OPB35_soil_group	3.776	1.003

The ecological indexes computed from the taxa abundance tables indicated the deeper sample as endowed with higher richness and evenness, as testified by the following values comparing, respectively, the shallow (−41 cm) and the deep (−279 cm) sample: Simpson 1-D: 0.969 *vs.* 0.979; Shannon H: 4.178 *vs.* 4.646; Evenness: 0.038 *vs.* 0.051; Chao-1 species number estimator: 1862 *vs.* 2112.

### ITS DNA sequencing

The analysis of the species-diagnostic amplified internally transcribed spacer eukaryotic target region yielded 67848 sequences from shallow sample (41 cm of depth) and 121666 sequences from the deep sample (279 cm of depth). After the annotation procedure, the number of OTUs at species rank level for the two samples was 310 and 291, respectively. Table 5 shows the proportional abundance distribution of all phyla accounting for a frequency higher than 1% in at least one of the two depths sampled. For the whole list refer to Supplementary Material dataset S1, spreadsheet, taxa abundance list.

**Table 5:** Eukaryotic OTUs % at species level (with cutoff >1%), sorted by decreasing abundance in the 41 cm-deep sample (top part of the table) or in the 279 cm-deep sample (bottom part of the table).

OTUs sorted by their abundance in the 41 cm-deep sample	Shallow sample	Deep sample
Ascomycota; Leotiomycetes; Helotiales; Helotiaceae; Neobulgaria; Neobulgaria_sp	20.571	0
Fungi; unidentified; uncultured_fungus	18.254	19.013
Fungi; LKM11	14.933	0.019
Ascomycota; Leotiomycetes	8.027	0.222
Ascomycota; Saccharomycetes; Saccharomycetales; Incertae; Sedis	4.983	0.075
Chytridiomycota	4.777	0.550
Ascomycota; Sordariomycetes	4.367	0.242
Basidiomycota; Agaricomycetes; Gloeophyllum	4.342	0.058
Basidiomycota	1.375	0.092
Metazoa; Mollusca; Bivalvia; Heteroconchia	1.344	0.127
Fungi; unidentified; uncultured_soil_fungus	1.325	0
**OTUs sorted by their abundance in the 279** **cm-deep sample**	**Shallow sample**	**Deep sample**
Fungi; unidentified; Antarctic_fungal_sp_GI911	0.295	26.408
Fungi; unidentified; uncultured_fungus	18.254	19.013
Basidiomycota; Microbotryomycetes; Sporidiobolales; Rhodosporidium_toruloides	0.010	18.732
Basidiomycota; Tremellomycetes; Bullera	0.125	10.379
Basidiomycota; Agaricomycetes; Russulales; Russulaceae; Russula; Russula_sp	0.681	4.818
Ascomycota; Archaeorhizomycetes; unidentified; Archaeorhizomycetes_sp	0.769	3.222
Basidiomycota; Tremellomycetes; Filobasidiales; Filobasidiaceae; Cryptococcus_friedmannii	0	1.770
Glomeromycota; Glomaceae	0.273	1.651
Basidiomycota; Tremellomycetes; Filobasidiales; Filobasidiaceae; Cryptococcus_albidosimilis	0.083	1.251
Basidiomycota; Agaricomycetes; Russulales; Russulaceae; unidentified; uncultured_Russula	0.165	1.134

**Figure 2:**
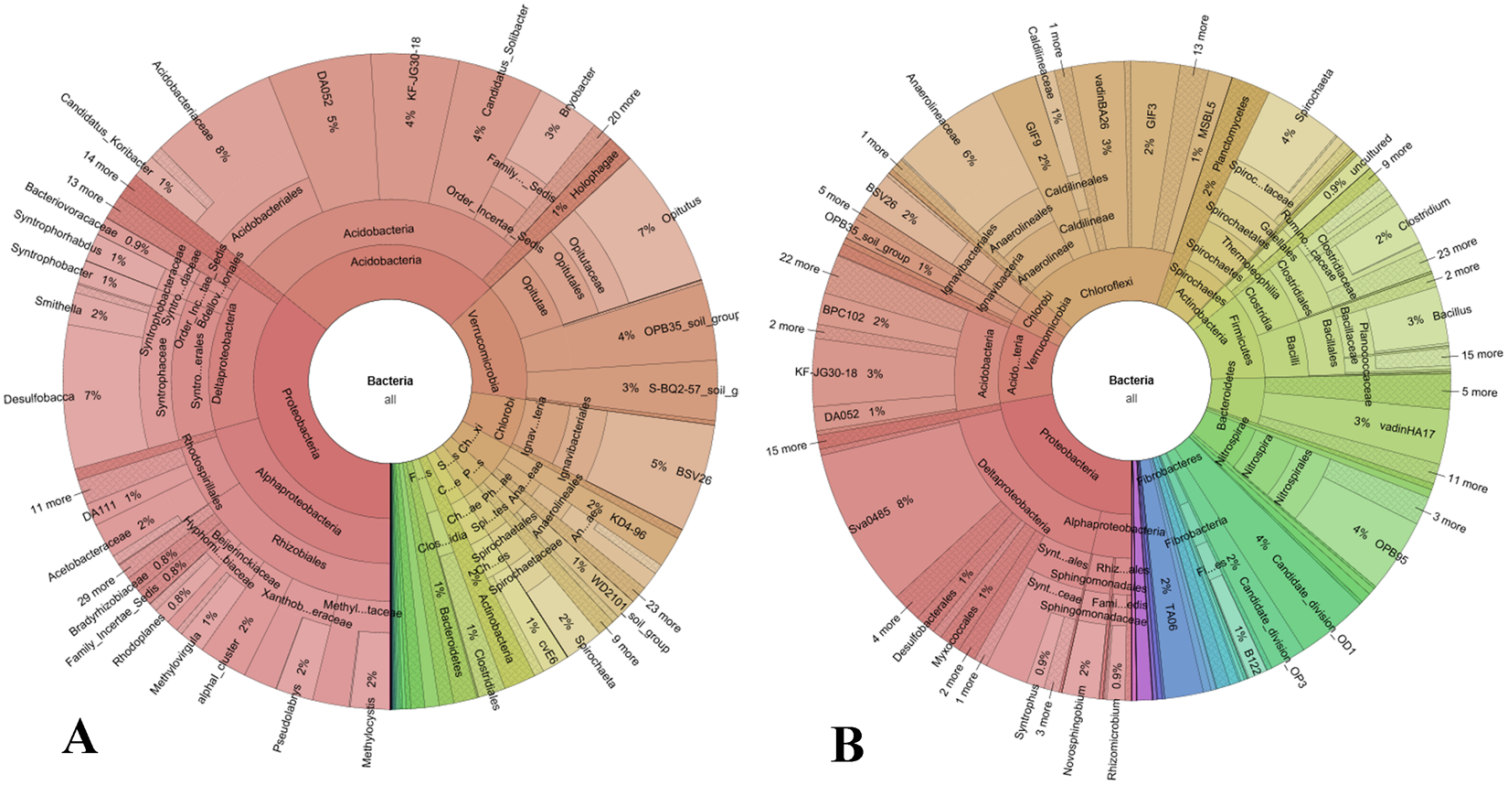
Krona pie chart plots of the bacterial communities proportional abundances (%) in the shallow (41 cm of depth) (**A**) and in the deep (279 cm of depth) (**B**) peat samples.

**Figure 3:**
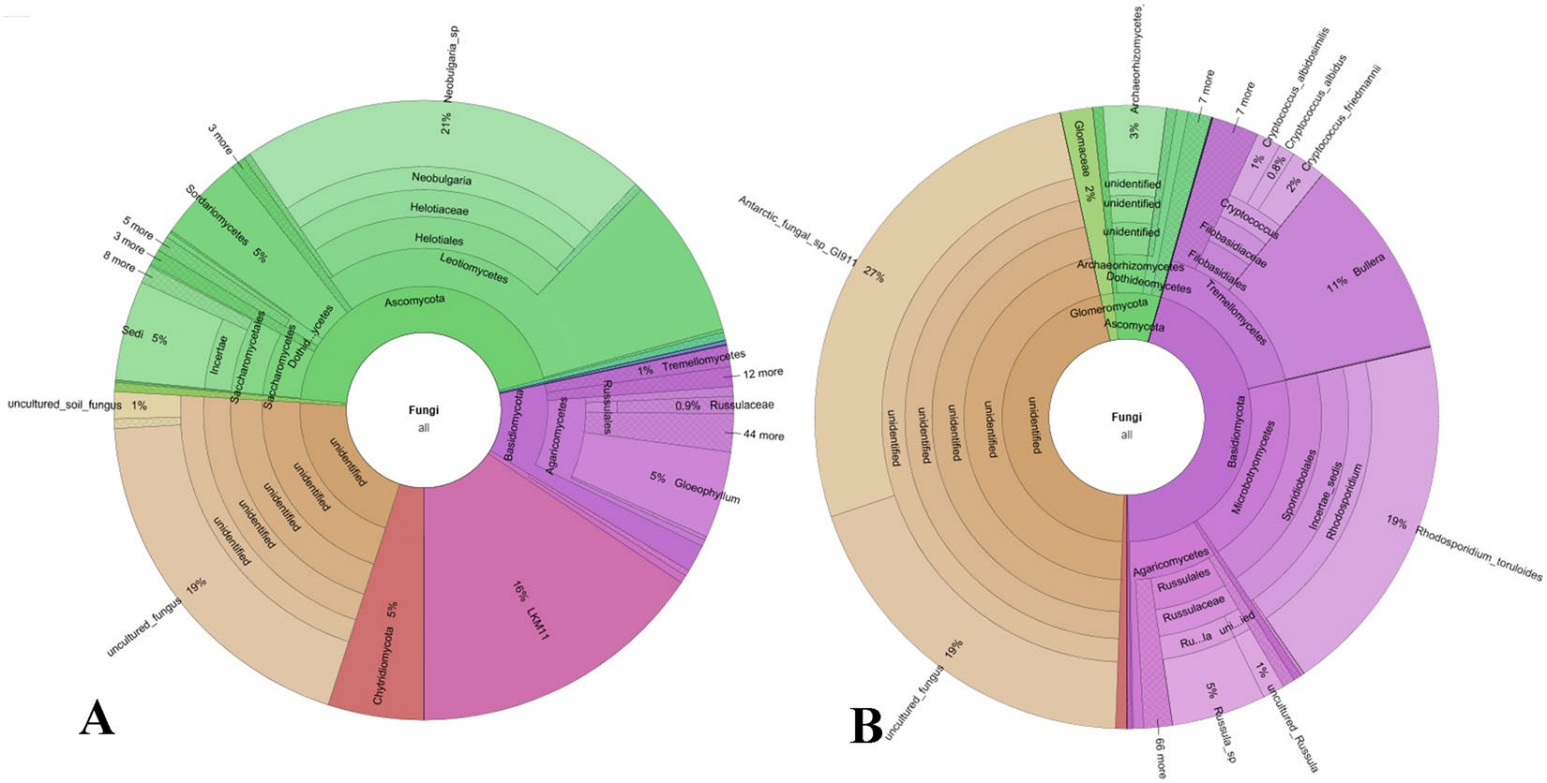
Krona pie chart plots of the fungal communities proportional abundances (%) in the shallow (41 cm of depth) (**A**) and in the deep (279 cm of depth) (**B**) peat samples.

To better visualize the main differences between the shallow and deep microbial communities, data were plotted using the Krona software^7^ and are shown as pie charts in Fig. 2 (bacteria) and Fig. 3 (fungi).

## Discussion

To properly frame the habitat for data interpretation, it is worth recalling that, upon chemical^8^, botanical^6^ and limnological analyses^9^, this lake was classified as lotic-lentic, i.e., a system that encompasses both the features of lake-type environments and those of river-type ones. The latter trait is due to a constant flow of karstic groundwater causing a continuous washout effect of dilution and a fast water removal. This in turn prevents the excessive accumulation of nutrients and ensures a consequent high transparency and oxygenation of the water surrounding the floating mire which qualifies the habitat as endowed with a good ecological rating. At the same time, due to the underground geochemistry, concentrations of dissolved phosphorus have been reported to reach values more similar to those of eutrophic and turbid lakes^8^.

The RealTime PCR analyses for the N cycle genes showed for all detected cases a higher gene abundance in the deeper sample. In particular, the bacterial (*amoA*) nitrifiers showed an advance in appearance of two threshold cycles in comparison with the shallow sample. An even greater difference was observed for the terminal gene of the denitrification pathway (*nosZ*) where the advance reached three cycles, testifying an eight-fold higher abundance. The nitrifying *amoA* Archaea genes were instead below the detection limit in both samples. That group is normally found in higher abundance with respect to the bacterial *amoA* when agricultural or overall non-submerged soils are commonly analyzed^10^; it is interesting to observe that such proportions appear more than reversed in this peculiar habitat.

In terms of abundance comparisons among the different reactions within the N cycle, it can be seen that the lowest gene presences (threshold cycle of 30 and higher) are for those activities requiring atmospheric gases supply in conspicuous amounts, such as O_2_ for nitrification (*amoA*) and N_2_ for N fixation (*nifH*). Conversely, genes encoding for reactions requiring overtly anoxic conditions as those of the denitrification (*nirK* and *nosZ*) are more abundant in both samples and culminate in the lower layer, in line with the submerged condition.

The fact that the nitrite reductase gene *nirK* is, within this complex, more represented than the nitrous oxide reductase *nosZ* is explainable by the notion that only a minority of denitrifiers are capable of carrying out the whole process to N_2_, while many would limit their anaerobic respiration to the reduction of nitrite to nitric oxide and lack the *nosZ* determinant^11^. Such disproportion appears less pronounced in the deep layer where the gap between *nirK* and *nosZ* resulted of only four cycles instead of seven, which is in agreement with the increased depth and the less oxic conditions that are more conducive to the complete reduction of the N forms.

As regards the taxa identification via DNA sequencing, first a consideration needs to be made regarding the experimental set up. The present analysis was carried out on samples from a single continuous core, which is the very same core whose chemical peculiarities have been recently reported in this journal^5^. We had considered the issue of replications which would have called for independent parallel sampling, i.e. collecting multiple peat cores through the site. In our study this posed, in first instance, a legislative constraint as the free-floating bog of Posta Fibreno, due to its renowned uniqueness, is a protected area in which any ordinary sampling, as well as trespassing, is forbidden. As a consequence, in order to carry out the present study, special and very limited permission to operate had to be negotiated with the competent Municipality and with the Regional Natural Reserve Managing Authority. For these reasons, it was for us out of question that data had to stem from more than a single core. Within that, given the low density of the peat, averaging around 0.035 g/cm^3^ in the top 2 m of depth and around 0.117 g/cm^3^ in the bottom 2 meters^5^, and the very low number of microbial cells per gram, it was not possible to obtain more than one usable sample for any given depth (the three replicates of the RealTime PCR in our data are technical and not biological ones).

Moreover, as a general and accepted rule in this discipline, assuming the perfect match among samples from different cores but corresponding to the same depths can be extremely misleading because of differential growth rates, variable compression during coring (possibly reflecting variations in density), micro-topographic differences, etc.^12^. This means that samples collected from two replicated cores and at the same depth may have a different age, although collected from the same bog at the same time. Therefore, a variability in microbial composition between replicates could be instead due to marked differences of deposition time, that could have thus carried over climate-related and event-related differences, which would have heavily biased the results.

For the above reported reasons, the choice of analyzing the microbial assemblages within the same continuous core was preferred, which allows comparisons of microbial community and chemical data stemming from the same core. Starting with the bacterial community, the 16S rRNA gene analyses showed that the shallow sample features a prevalence of Proteobacteria (mostly of the alpha class), Acidobacteria and Verrucomicrobia, which makes it more reminiscent of “*terra firma*” soils if compared to the deep sample where the Proteobacteria are mostly of the delta class, the Chloroflexi are peaking and the Firmicutes result the next most abundant phylum followed by Bacteroidetes. Both Firmicutes and Bacteroidetes are typically found in environments hosting processes of organic matter decomposition, in different climatic conditions^13–15^.

In the lower (279 cm deep) sample, the evident peak of Nitrospirae (*amoA* eubacteria nitrifyier) is in agreement with the RealTime results discussed above. The fact that an oxidative reaction as nitrification has its actors more abundant at lower depth where also denitrification is more active is not in contrast with current knowledge of the N cycle as the product of nitrification (nitrate) is the substrate of denitrification and the spatial heterogeneity of soils provides different redox conditions within a small scale. A further instance of nitrification potential at the lower depth is the occurrence of the Archaea Thaumarcheota. Their non-detectability result in the RealTime experiment could be in part due to their variability in the target sequences with respect to the primer consensus. The Euryarcheota group, also more abundant in the deep sample, includes different methanogenic and extremophilic microorganisms. A high abundance of the rare phylum Candidate Division OD1 was noticeable, as this taxon has been previously observed in boreal latitude lakes, where it is implicated in the carbon cycle within methanogenic environments^16^.

When looking at the finer taxonomy resolution level, in the shallow sample the top scoring OTU (>7%) is represented by the genus *Desulfobacca*, a sulphate reducer observed in aquatic sediments^17^, followed by *Opitutus*, reported in rice fields and paddy anoxic soils^18^ and by various members of the family Acidobacteraceae which are degraders of organic matter in wet environments^19^.

The occurrence of Acidobacteria in the shallow layer and that of methanogenic archaea in the deeper one is consistent with results found in a Swiss ombrotrophic peat bog analyzed by DGGE^20^.

Interestingly, also in the deep sample, the top OTU is a sulfate-reducing Deltaproteobacteria member (Sva0485) and represents 7% of the total OTUs, but its identity is different from that of the one that dominates the shallow sample, indicating a net taxon shift within the same community guild. Next in abundance in the deep sample is a member of the Anaerolineaceae (Chloroflexi phylum) which degrade hydrocarbons in syntrophic association with methanogens^21^.

Seeking for other similarities between this work and other microbial analyses of peat-forming context, Dedish and coworkers^22^ reported that, consistently with the present results, in the dominant phylum Proteobacteria only Alpha- and Delta- classes were found by analyzing 16S rRNA gene libraries of cloned amplicons. A different study also reported the presence of methanogens in mires^23^. In our case, it is worth commenting that the presence of taxa as Anaerolineaceae, associated with methanogenic ones, and Euryarcheota, which are, as expected, found in the deeper and less oxic layer, could be fueled by the naturally occurring flow of CO_2_ from underground sources reported for the Posta Fibreno lake^8^.

There are two principal kinds of peatlands: minerogenic peat deposits such as fens, swamps, and marshes, which are fed mainly by surficial and groundwater and generally show a circumneutral pH, and ombrogenic bogs, which are nourished exclusively by rainwater and dust, and generally show an acidic pH^24^. The free-floating island of Posta Fibreno is a peculiar formation whose features have been interpreted as somewhat hybrid between the two above types of mires. In fact, Zaccone *et al*.^5^ classified this free-floating island as a transitional mire with a secondary ombrotrophic local dominance induced by buoyancy. The top 100-cm layer, consisting almost exclusively of undecomposed *Sphagnum*, is fed by rains and generates a bog-like environment (pH ~5.5), whereas the bottom 300-cm, being today in karstic/lotic conditions, features a pH around 7. We probed the two distinct representative layers of this unique configuration also to verify the compliance to this dual facet in terms of microbial assemblages. Literature reports comparing the microbiology of bogs and fens showed that (i) bacterial diversity richness results higher in fens, and (ii) that bogs are dominated by Acidobacteria while fens show consistent numbers of Firmicutes^25^. As regards Archaea, the same authors report fens as considerably richer in hydrogenotrophic methanogens^25^. It is interesting to remark that all these conditions are consistent with the findings that we observe in the respective upper and lower portions of the Posta Fibreno free-floating island. The 279 cm-deep sample (PFB4#16-17) had in fact higher values of the Simpson, Shannon Evenness and Chao-1 indexes; the shallow layer (PF2#46) was also dominated by Acidobacteria while its lower counterpart had about 7x higher levels of Firmicutes. We also found a nearly 10x prevalence of the hydrogenotrophic methanogens in the deeper sample. These consistencies support the view of the Posta Fibreno island as a system encompassing the transition from minerotrophic to ombrotrophic conditions over time^5^.

The Eukaryotes ITS sequencing analysis, as the fungal counts are concerned, highlighted major differences between the two layers with a reciprocally inverted dominance of Ascomycota *vs.* Basydiomycota; the former represent 46% of the ITS OTUs in the shallow sample and a mere 6% in the deep one, whereas the latter feature 45% in the deep sample and 12% in the shallow one. Moreover, also the detected species are rather different: the Basydiomycota of the deep sample are mainly represented by *Rhodosporidium toruloides* (19% of all fungi in the sample), a red oleaginous yeast known to accumulate triacylglycerol lipids from lignocellulosic biomasses^26^ and by *Bullera* sp. (11%). The main species in the shallow sample belong to the non-dominant Agaricomycetes, which are mostly known as associated to wood decay.

The dominance of Ascomycota in the upper sample is mostly accounted for by the genus *Neobulgaria*, belonging to the Helotiaceae family. The fungi of this family are mostly saprotroph on herbaceous or woody plants. In the deep sample, on the contrary, this taxon (dominating the fungi of the upper layer) was not detected and the small contingent of Ascomycota was mostly represented by the wood decomposing Archaeorhizomycetes.

In the shallow sample, a relative abundance of Chytridiomycota (5%) is observed, which are known as fungi equipped with flagellated zoospores and exploiting shallow water habitats or wet soils.

LKM11, very abundant in the shallow sample (16% of all fungi), belongs to the specific environmental clade Rozellida, which is the deepest branch of true fungi so far identified^27^.

The dominant taxon of the deep layer (26%) features species-level identity with an isolate from the accretion ice in the Antarctic lake Vostok^28^.

All considered, the in-depth microbiological characterization of the relictual, free-floating island of Posta Fibreno has delineated some microbial features amenable to processes that are shared with other mires and sites located in completely different environments, including extreme latitudes and colder climates. Such consistencies include: (a) an organic matter supply from woody type vegetation in an oxygen-limiting high-water table situation; (b) the consequent generation of slowly dissolved organic carbon compounds, including one-carbon atom molecules conducive to microbial methylotrophy and, in deeper layers, to methanogenesis; (c) a supply of CO_2_ resulting either from heterotrophic respiration fueled by oxygen trapped in air pockets in the floating peat layers and/or directly by geochemical escape from karstic sinkholes at the basin bottom.

In the community composition some trends appear to point at an unusual concurrent species convergence between this sub-Mediterranean site and far Nordic or circumpolar environments. Although this instance would require more robust comparisons to be corroborated, it could lead to hypothesize that the variables ultimately shaping the microbial interfaces of our landscapes are primarily process-driven in spite of the differences conferred by macroclimatic and geographical constraints. These hypotheses are also in agreement with botanical reports^6^ who defined the Posta Fibreno area as a refuge site for boreal plant species.

The microbial analysis hereby commented offers further issues to be dissected; as in most environmental contexts, the culture-independent presence of DNA from any microbial taxon is not allowing to distinguish whether it represents an archived biological record from the same age of the layer or a colony that could have reached the place any time from its origin to present. In this respect, it is not possible to discern whether the presently described communities should be regarded as relict or modern but the firm point is the possibility to compare their taxonomy with that of the ones occurring in other environments and to verify which processes do the respective habitats have in common.

The present study, besides providing an insight on the hitherto unexplored microbiology of a free-floating mire, offers hints for more extended ecological analyses. For example, some similarities between this Mediterranean mire and Nordic environments individuate a possible principle of minimum common denominator between habitat divergence and biotic community convergence. This example can be of use to further explore the hierarchical order upon which physical and chemical effectors impinge on microbial communities composition across environments. Their taxonomical composition and the underlying physiology may provide useful elements to better understand the functioning of this natural ecosystem and/or its formation, especially considering that much of the floating island formation and evolution, as well as the changes of the surrounding areas, are recorded in its peat deposit.

## Methods

### Sample collection and preparation

A complete, 400-cm deep peat profile was collected on 18^th^ July 2012 from the central domed area of the Posta Fibreno free-floating mire (41° 41′ 41.8″ N; 13° 41′ 30.3″E; 290 m a.s.l.), where the surface peat layers are clearly elevated up to ~1 m beyond the edge of the isle. The uppermost 100-cm core was collected using a Wardenaar sampler (15 × 15 × 100 cm), whereas the remaining 300-cm were collected using a Belarus corer (semi-cylindrical peat sections, 50 × 10 cm).

Once collected, peat cores were wrapped in polyethylene cling film, placed in specifically-built boxes, brought to the lab and frozen at −18 °C. Peat cores were then cut while frozen in 1-to-2 cm slices using a stainless steel band saw, in the ultra-clean SWAMP lab (University of Alberta, Edmonton, Canada). The edges (~1 cm) were trimmed away from each slice.

Two samples were selected for this study: the first one (shallow sample, PF2 #46), collected at *ca.* 40 cm of depth, consists of almost undecomposed *Sphagnum palustre* material, whereas the second one (deep sample, PFB4 #16–17), collected at *ca.* 280 cm of depth, consists of silty peat rich in Graminaceae, Cyperaceae and root material (Fig. 1d). Main physical and chemical properties of these samples are summarized in Table 6.

**Table 6:** Main physical and chemical properties of the two peat samples (data from^5^).

	Avg. depth (cm)	Dry density (g/cm^3^)	Ash (%)	Water content (%)	Gravimetric water content (g_water_/g_peat_)	pH	C_org_	N_tot_	δ^13^C	δ^15^N
PF2 #46 (shallow)	−41	0.044	4.2	95.3	20.5	4.5	43.6	0.55	n.d.	n.d.
PFB4 #16–17 (deep)	−279	0.120	10.3	90.9	10.0	7.0	45.3	2.78	−27.1	3.0

### DNA extraction and purification

DNA extraction from peat samples was performed on air dried material (250 mg) using a Genomic DNA Soil kit from Macherey Nagel INC. Bethlehem, PA USA as recommended by the manufacturer. The protocol involved a mechanical lysis with beads and a lysis based on SDS. The lysate was purified by passage through a Nucleospin Inibitor Removal Column, and eluted in 100 μl. Three independent isolations were performed for each soil sample. Purified DNA was then stored at −20 °C.

### Quantification of nucleic acids concentration

After nucleic acids extraction, the concentration of DNA was calculated with the Quant-iT^TM^ PicoGreen® dsDNA Assay kit (Invitrogen, Eugene, Oregon, USA). For the quantification, 3 µl of soil DNA samples were diluted in 350 µl TE buffer (diluted from 200x solution: 200 mM Tris-HCl, 20 mM EDTA, pH 7.5), and a standard DNA solution (provided in the kit) was diluted at the concentrations of 500, 100 and 10 ng/µl to permit the construction of a standard curve for the exact quantification. 100 µl PicoGreen (diluted to 1 x concentration in TE buffer) were dispensed in a black 96 well plate together with 100 µl of the samples and the standard DNA (three technical replicates for each sample). The concentration was verified by a Spectrofluorimeter (SpectraFluor, TECAN, Männedorf, Switzerland) using as excitation and emission wavelengths 485 and 535 nm, respectively, as well as spectrophotometrically on a Nanodrop (Thermo Scientific, Tewksbury, MA, USA).

### Amplification Primers

Primers were supplied by Eurofins MWG Operon (Ebersberg, Germany) or by Biomers (Ulm, Germany) and dissolved in MilliQ sterile water to a final concentration of 100 pmol/µl. Primers were stored at −20 °C. Sequences of the primers used and their references are shown in Table 7.

**Table 7:** Gene specific primers used in this work. The 16S rRNA gene and ITS sequencing primers were the ones described by the manufacturer (Illumina Inc.) for the metagenomic library preparation guide # 15044223-b.

Primer Name	Sequence 5′ –>3′	Gene	Reference
Arch-amoAF	STAATGGTCTGGCTTAGACG	Archaeal *amoA*	29
Arch-amoAR	GCGGCCATCCATCTGTATGT	Archaeal *amoA*	29
amoA1F	GGGGTTTCTACTGGTGGT	Bacterial *amoA*	30
amoA2R	CCCCTCKGSAAAGCCTTCTTC	Bacterial *amoA*	30
nosZF	CGYTGTTCMTCGACAGCCAG	*nosZ*	31
nosZR	CATGTGCAGNGCRTGGCAGAA	*nosZ*	31
nirK876F	ATYGGCGGVCAYGGCGA	*nirK*	32
nirK1040	RGCCTCGATCAGRTTRTGGTT	*nirK*	32
nifHF	AAAGGYGGWATCGGYAARTCCACCAC	*nifH*	31
nifHR	TTGTTSGCSGCRTACATSGCCATCAT	*nifH*	31

The 16S rRNA gene and ITS sequencing primers are the ones specified by the manufacturer, available at https://www.illumina.com/content/dam/illumina-support/documents/documentation/chemistry_documentation/16s/16s-metagenomic-library-prep-guide-15044223-b.pdf.

### Quantitative Real Time PCR

Quantitative Real Time PCR (qRT-PCR) technique was used to quantify the levels of functional genes of the N cycle. Peat samples were processed in automation using a Tecan Freedom Evo 100 robot. The assay was carried out with Power SYBR® Green PCR Master Mix (Applied Biosystems) in a 7900HT Real Time PCR System (Applied Biosystems) using 384-wells plates. Gene-specific primers (listed in Table 7) were used to amplify the desired specific genes. The PCR conditions specific for each given pair are reported in Table 8. Three technical replicates per sample were run.

**Table 8:** Conditions for the different primers in the RealTime PCR.

Gene	Amplicon’s length	Primers’ concentration	Annealing Temperature	Extension time
Archaeal *amoA*	635 bp	0.5 µM	57 °C	45 s
Bacterial *amoA*	~500 bp	0.5 µM	57 °C	40 s
*nosZ*	706 bp	0.5 µM	56 °C	45 s
*nirK*	~160 bp	0.5 µM	58 °C	30 s
*nifH*	432 bp	0.5 µM	53 °C	45 s

### DNA sequencing

For each sample, 500 ng of purified DNA with an OD260/280 >1.8 and 260/230 close to 2 was used. DNA quantification was carried out by fluorescence (Picogreen) and quality check was performed an Agilent Tapestation. Libraries were constructed following the Illumina amplicon sequencing protocol using primers for the detection of the V3-V4 16S rRNA genomic region for bacteria and ITS for eukaryotes. Sequencing was performed on an Illumina MiSeq platform with a 2 × 300 bp protocol.

Raw reads quality was checked using FastQC v0.11.2. Illumina raw sequences were trimmed using Trimmomatic v0.32 with a minimum base quality 20 (Phredscale) over a 6 bases sliding window. Only sequences above 36 nucleotides in length were included into downstream bioinformatics analysis. The reference database was created merging Qiime formatted ITS1 (its109022014, 97% clustered version) database with the 16S rRNA gene + 18S rRNA gene Qiime formatted SILVA (Silva111, 97% clustered version) database. The reference databases taxonomies were adapted to Qiime taxonomy standards uniforming to the 7 main taxa ranks (superkingdom, phylum, class, order, family, genus, species). For the metabarcoding following a reference-based outpicking strategy, for each sample raw trimmed reads were aligned against the reference database using bwa mem v0.7.9. Quantification was performed using Samtools. OTUs tables were created using custom scripts. OTUs tables in BIOM file format were generated using Qiime Biom Convert software. For data visualization, graphical interactive html taxonomy abundance piecharts were generated using KronaTools v2.4. Ecological diversity indexes were computed using the Past software v.3.14^33^.

## Electronic supplementary material

Dataset 1
